# A Manual of Obstetrics

**Published:** 1897-09

**Authors:** 


**A Manual of Obstetrics.** By W. A. NEWMAN DORLAND, M.D. Pp. 760. London: Henry Kimpton. 1896.

This is an interesting and useful work, since it embodies fairly completely the practice and teaching of the American school of obstetricians. It is well arranged and well illustrated, the various sections being placed in their physiological and corresponding pathological sequence, but the description of the development of the ovum might have been treated more fully in the light of the large amount of research work that has lately been done on this subject.

The descriptions of physiological pregnancy and labour are very full and clear; the pathological conditions which occur in connection with these are well described, and the treatment clearly indicated. Ectopic gestation is dealt with very fully and carefully. The author considers that Tait and others, who look upon all cases of ectopic gestation as primarily tubal, are mistaken, but at the same time acknowledges the extreme rarity of primary ovarian or abdominal extra-uterine pregnancy. He describes very fully the hemorrhages met with in parturition and the puerperium, but is at variance with the opinion of many British authorities on some points. For example, he recommends *accouchement forcée* in cases of accidental hemorrhage—a method of treatment which is by most English authorities considered a bad one, except under certain exceptional conditions. In dealing with eclampsia he recommends as the most successful methods in various cases, chloroform narcosis, venesection, hot wet or dry packs, and the injection of veratrum viride, and omits all mention of the injection of morphia, a treatment which has been advocated in Europe for the last four or five years, and which has, in the hands of its introducers and others, proved most valuable. The method of treatment by veratrum viride injected subcutaneously appears to us to be verging on the heroic: the induction of such an amount of cardiac depression as to necessitate the administration of alcohol by the rectum or ether subcutaneously in large doses would scarcely seem desirable, the difficulty of limiting the downward limit of cardiac depression being no small one.

The description of the septic affections of childbed is very good, and treated in detail to an extent which the importance of the subject fully warrants. He classes under the head of sapræmia both those conditions which are usually differentiated as septicæmia and sapræmia, but at the same time lays down the proposition that the general septic poisoning in association with which pathogenic germs are found in the blood is almost invariably preceded by local decomposition and absorption of chemical products of microbic growth, and bases on this, which undoubtedly is true in the larger number of cases, a strong and convincing argument for the early and energetic treatment, and especially for the early recognition, of cases of puerperal sepsis. The name used is unimportant, provided correct principles of treatment based on pathological foundations are advanced, and the emphasis of his remarks on this condition alone would render the work a valuable one.

Very useful hints are given with regard to some of the minor but most annoying ailments which occur in connection with the pregnant woman and the infant, the treatment of which in the case of the newly qualified practitioner has usually to be evolved from his inner consciousness.

